# Echoes of the mind’s eye: Reciprocal crossmodal interaction between auditory and visual processing

**DOI:** 10.1016/j.isci.2026.114990

**Published:** 2026-02-12

**Authors:** Xiaoyu Tang, Ting Zhang, Jiaying Sun, Sa Lu

**Affiliations:** 1 School of Psychology, Liaoning Collaborative Innovation Center of Children and Adolescents Healthy Personality Assessment and Cultivation, Liaoning Normal University, Dalian 116029, China; 2 School of Foreign Languages, Ningbo University of Technology, Ningbo 315211, China

**Keywords:** classification description, biological sciences, neuroscience, sensory neuroscience

## Abstract

Understanding how the brain integrates information across senses is a fundamental challenge. We investigated the mechanisms underlying reciprocal yet temporally asymmetric auditory-visual interactions using high-density EEG during an auditory spatial attention task with auditory-only (A) and audiovisual (AV) stimuli. In the AV condition, sounds were paired with a central, task-irrelevant visual input. Participants attended to sounds on one side (attended) and ignored the other (unattended). Results showed that the visual input affected auditory processing through initial stimulus-driven suppression, reflected in the eliminated auditory contralateral sensory bias (selection negativity, 220–320 ms) and attenuated fronto-temporal connectivity and auditory contralateral occipital positivity (ACOP, 300–500 ms). Subsequently, auditory-to-visual cross-modal attentional spreading exhibited attention-dependent facilitation, emerging over occipital (300–600 ms) and centro-parietal (500–600 ms) regions. These findings support a competitive-facilitative framework in which stimulus-driven suppression precedes goal-driven facilitation, providing a key temporal constraint for computational and neurocognitive models of crossmodal integration.

## Highlights

• Task-irrelevant visual input initially suppresses auditory spatial attention
• Early suppression eliminates auditory contralateral bias and fronto-temporal coupling
• Later auditory-to-visual attentional spreading shows goal-dependent facilitation
• Multisensory integration follows a competitive-to-facilitative temporal framework

## Graphical abstract

**Figure:**
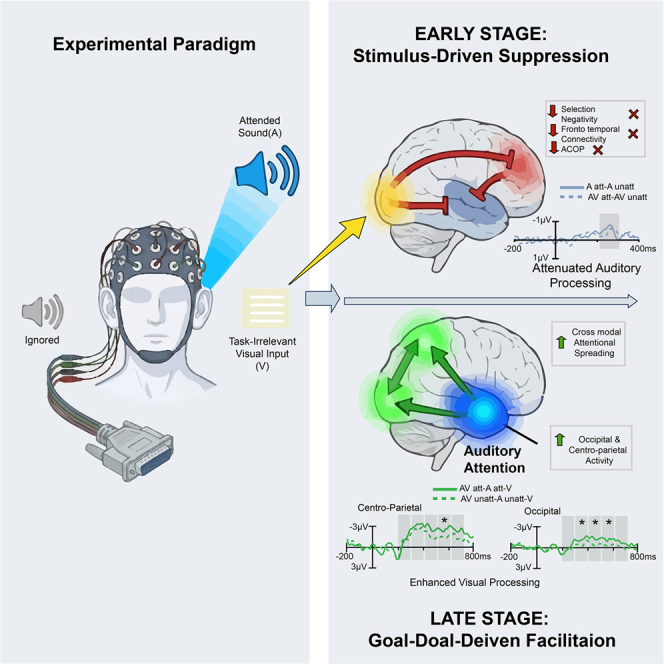

## Introduction

Perception of the external world emerges from the brain’s ability to dynamically integrate information across multiple sensory modalities. Among these, the interaction between auditory and visual systems plays a pivotal role in shaping conscious experience. Rather than operating independently, these sensory modalities interact continuously, influencing the content, timing, and salience of perceptual representations.^1^^,^^2^^,^^3^^,^^4^^,^^5^ Extensive evidence shows that visual inputs can alter the perception of spatial and temporal attributes of sounds, while auditory cues often enhance visual detection and discrimination.^6^^,^^7^^,^^8^^,^^9^^,^^10^ However, despite this rich literature, research has often remained compartmentalized by examining vision influencing audition and audition influencing vision in isolation, thereby leaving their integrated reciprocal dynamics and potential temporal asymmetry insufficiently understood.

A large body of work has demonstrated that auditory stimuli can modulate visual cortical activity through rapid, automatic, and crossmodal pathways.^11^^,^^12^^,^^13^^,^^14^ A typical electrophysiological indicator of this is auditory contralateral occipital positivity (ACOP), a positive ERP component observed over occipital electrodes contralateral to the sound source, typically occurring within the 200–500 ms time window.^11^^,^^14^^,^^15^^,^^16^^,^^17^ ACOP has been conceptualized as a purely stimulus-driven response, reflecting automatic engagement of visual spatial attention by salient, lateralized auditory input.^14^^,^^15^^,^^17^ Some studies found that ACOP can be modulated by higher-level contextual factors such as task demands or audiovisual congruency: spatial alignment and short stimulus onset asynchrony (SOA) enhance ACOP, whereas incongruence impairs or delays it.^16^^,^^18^ However, it remains unclear whether temporally synchronized but centrally presented, task-irrelevant visual stimuli can nonetheless modulate ACOP. We reasoned that although temporal synchrony may facilitate integration, a spatially uninformative visual stimulus could compete with the lateralized auditory signal for neural resources in the visual cortex. Based on multisensory integration frameworks, such spatially mismatched inputs are likely to engage suppressive gain-control mechanisms rather than facilitatory integration.^19^^,^^20^ It follows that a stimulus-driven suppressive interaction may manifest as a reduction in the ACOP amplitude when a central, task-irrelevant visual stimulus is presented concurrently.

Complementarily, attention directed toward a visual event can influence concurrent auditory processing. A well-documented phenomenon is visual-to-auditory crossmodal attentional spreading, in which attention allocated to one modality (e.g., vision) enhances the processing of concurrent stimuli in another (e.g., audition), even when the auditory is task-irrelevant.^21^^,^^22^^,^^23^^,^^24^^,^^25^^,^^26^^,^^27^^,^^28^^,^^29^^,^^30^^,^^31^^,^^32^^,^^33^^,^^34^ This effect is typically indexed by a late-onset (>200 ms), sustained, negative-polarity ERP linked to enhanced auditory processing. Such crossmodal attentional spreading causes initially unattended auditory components of audiovisual stimuli to be drawn into the attentional spotlight, facilitating their processing. Despite robust evidence for visual-to-auditory crossmodal spreading, whether auditory spatial attention can similarly modulate visual processing remains unresolved.

Prior studies indicate that auditory-driven crossmodal influences—especially during late-stage multisensory integration—are typically weaker and less reliable than those observed in other modality pairings.^35^^,^^36^ This observed asymmetry necessitates a more nuanced perspective on the very concept of reciprocity in crossmodal research. A purely symmetrical account of bidirectional influences may be insufficient to capture the complex dynamics of audiovisual integration. Instead, we propose a theoretical reconceptualization: that robust reciprocal interaction can emerge from the concurrent operation of distinct, functionally asymmetric neural pathways operating within a unified attentional context. This framework shifts the critical question from merely whether auditory-to-visual effects are truly reciprocal to elucidating the specific nature of these interactions. Specifically, it directs inquiry toward whether they reflect intrinsic differences in spatial precision, temporal dynamics, and attentional control across sensory modalities^37^^,^^38^^,^^39^ and how distinct types of reciprocal influences co-occur and interact. A key source of current ambiguity is the unresolved issue of whether auditory-to-visual interactions are primarily stimulus-driven (e.g., as indexed by the ACOP) or goal-directed.

Building on these phenomena and literature gaps, this study seeks to address the following theoretical question: How do reciprocal audiovisual interactions operate temporally and asymmetrically? We specifically test the overarching hypothesis that these crossmodal influences are dissociable in time and mechanism, characterized by an initial, stimulus-driven suppression from vision to audition and a subsequent, goal-directed facilitation from audition to vision. Accordingly, we hypothesize that task-irrelevant visual stimuli will suppress auditory processing, as reflected in reduced ACOP and attenuated auditory selection negativity in the audiovisual condition compared to the auditory-only condition, representing stimulus-driven suppression. In contrast, we expect auditory spatial attention to enhance visual processing, as indicated by a sustained negative difference (extracted visual minus visual-only) that was larger for attended than unattended auditory streams, indicating goal-driven cross-modal attentional spreading. These findings would represent stimulus-driven suppression in the case of visual-to-auditory interactions and goal-driven facilitation in the case of auditory-to-visual interactions. We further hypothesize that these interactions will exhibit temporal asymmetry, with stimulus-driven suppression (visual-to-auditory) occurring first and goal-driven facilitation (auditory-to-visual) occurring subsequently. Furthermore, complementary functional connectivity analyses will allow us to investigate whether the predicted crossmodal interactions are associated with distinct patterns of communication between auditory, visual, and attentional control regions.

**Figure 1:**
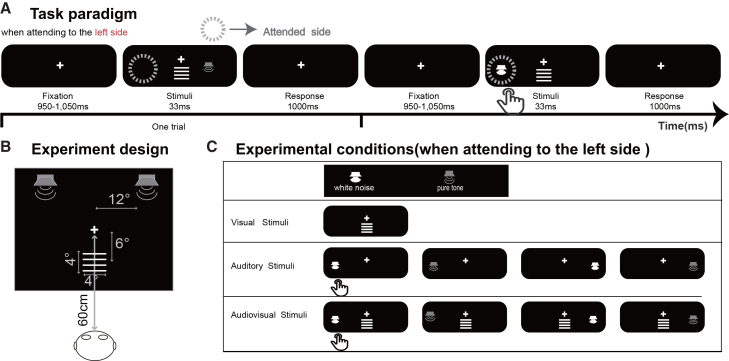
Example of the stimuli and experimental procedure (A) Task paradigm shown for trials in a block when the attended side was left. The task for subjects was to press a button in response to hearing white noise at the to-be-attended spatial location (left or right side) while ignoring all sounds at the unattended side and all drawings if delivered. (B) The size and position of the stimuli are shown in the upper left panel. (C) Experimental conditions. The visual stimulus could be the white horizontal square wave grating, which always came from the center location. The auditory stimulus, including the target stimulus of white noise and the non-target stimulus of pure tone, was presented randomly to the left or right auditory field. The central drawings (V) and unilateral sounds (A) could be either presented alone or presented synchronously (AV).

To test these hypotheses, we employed high-density EEG recordings with an endogenous auditory spatial attention paradigm while presenting spatially neutral, task-irrelevant visual stimuli (see Figure 1). Two experimental conditions were tested: auditory-only (A) and audiovisual (AV), under both attended and unattended attention states. Participants will attend to auditory stimuli presented from one side (left or right) while ignoring auditory stimuli presented from the opposite side. This specific design was strategically adopted to dissociate two fundamental classes of crossmodal mechanisms within a unified experimental framework. By maintaining visual stimulation as both task-irrelevant and spatially neutral, we aimed to isolate its automatic, stimulus-driven influence on concurrent auditory processing. Simultaneously, by systematically directing endogenous attention within the auditory modality, we sought to capture any goal-driven, top-down modulation exerted by auditory spatial attention on visual cortical processing. This design allowed us to disentangle two fundamental crossmodal mechanisms: visual-to-auditory modulation indexed by the auditory spatial attention effects and ACOP and auditory-to-visual spreading reflected in the sustained negative difference ERP within the same experimental paradigm. By characterizing these distinct yet complementary mechanisms, our study not only clarifies how attention dynamically coordinates information across sensory modalities but also advances a more nuanced conceptual framework for understanding reciprocity in audiovisual interactions.

## Results

### Behavioral results

The ACC of all participants was 97.72%. The difference in the ACC between the auditory and audiovisual conditions was not statistically significant (*t*_(24)_ = 1.502, *p* = 0.146, *d* = 0.300; auditory targets: *M* = 98.20%, *SE* = 1.96; audiovisual targets: *M* = 97.24%, *SE* = 2.98). Additionally, a paired-sample *t* test comparing RTs to auditory and audiovisual conditions revealed no significant difference in RTs (*t*_(24)_ = 0.395, *p* = 0.696, *d* = 0.079; auditory targets: *M* = 522 ms, *SE* = 68.51; audiovisual targets: *M* = 524 ms, *SE* = 74.00). (see Table 1).

**Table 1:** The overall performance of participants under the A (auditory) and AV (audiovisual) conditions

modalities	ACC (%)	RT (ms)
A (*M* ± *SE*)	98.20 ± 1.96	522 ± 68.51
AV (*M* ± *SE*)	97.24 ± 2.98	524 ± 74.00

Notes: ACC (accuracy, %), RT (reaction time, ms).

### ERP results

#### Spatial attention effect

**Figure 2:**
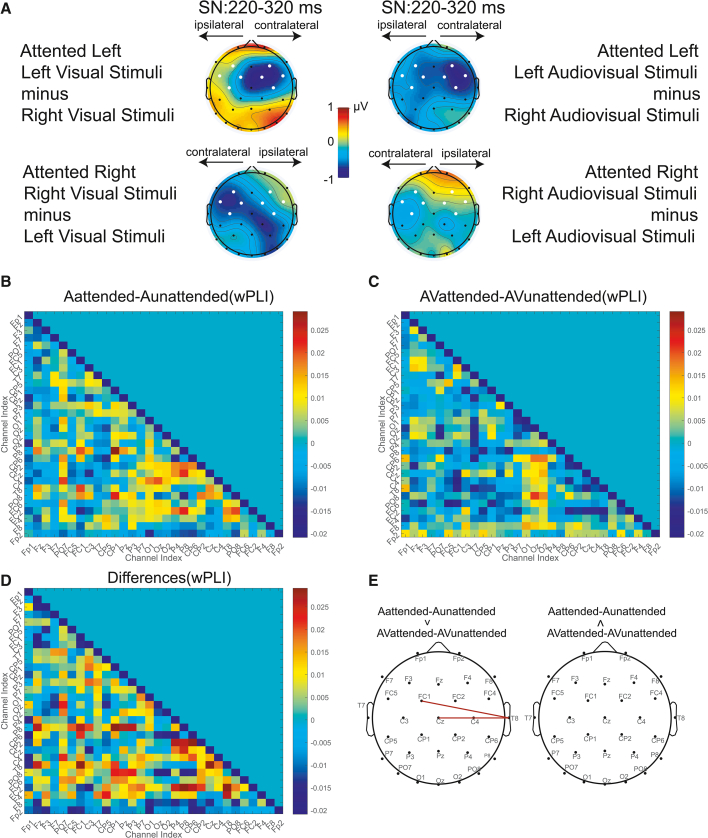
Effects of spatial attention on ERP and functional connectivity (A) Topographic maps show the scalp distributions for visual stimuli (left panels) and audiovisual stimuli (right panels). For the left-attended condition, the maps are computed as left visual/audiovisual stimuli minus right stimuli; for the right-attended condition, they are computed as right visual/audiovisual stimuli minus left stimuli. Warmer colors indicate more positive amplitudes, and cooler colors indicate more negative amplitudes, revealing the contralateral SN patterns under visual and audiovisual stimuli. The white dots on scalp topographies depict the fronto-central ROI (FC1, FC2, FC5, FC6, F3, F4, C3, and C4) over which each component was measured. (B) Functional connectivity matrices for attended vs. unattended auditory conditions, computed using the weighted phase lag index (wPLI; 30 × 30 channels). Diagonal elements are set to zero. (C) Functional connectivity matrix for attended vs. unattended audiovisual conditions. (D) Difference matrix comparing the connectivity contrasts of auditory and audiovisual conditions (attended vs. unattended). (E) Topographic map highlighting electrode pairs showing significant wPLI connectivity after FDR correction. The attended-unattended contrast revealed stronger functional coupling among the frontal (FC1), central (Cz), and right temporal/auditory (T8) regions in the auditory condition than in the audiovisual condition.

Separate three-way repeated-measures ANOVAs were conducted on the mean amplitudes of the SN components for the auditory and audiovisual conditions, with factors of attention location (left, right), stimulus location (left, right), and electrode location (left, right). For the auditory condition (see Figure 2A), the analysis revealed a significant two-way interaction between attention location and stimulus location (*F*_(1,24)_ = 7.509, *p*_*FDR*_ = 0.032, η_p_^2^ = 0.238). Simple effects analysis showed that when the stimulus appeared on the left, the SN amplitude was significantly more negative when attention was directed to the left (*M* = 2.217 μν, *SE* = 0.442) compared to the right (*M* = 2.926 μν, *SE* = 0.523; *p* = 0.028). The analysis also revealed a significant two-way interaction between stimulus location and electrode location (*F*_(1,24)_ = 20.632, *p*_*FDR*_ < 0.001, η_p_^2^ = 0.462). Simple effects analysis showed that when the stimulus was on the left, the amplitude was significantly more negative over the right hemisphere (*M* = 2.309 μν, *SE* = 0.492) than over the left hemisphere (*M* = 2.834 μν, *SE* = 0.442; *p* = 0.006). The main effects of attention location (*F*_(1,24)_ = 0.917, *p*_*FDR*_ = 0.418), stimulus location (*F*_(1,24)_ = 0.009, *p*_*FDR*_ = 0.924), and electrode location (*F*_(1,24)_ = 2.796, *p*_*FDR*_ = 0.222) were not significant. The interaction between attention location and electrode location was not significant (*F*_(1,24)_ = 0.000, *p*_*FDR*_ = 0.989), and the three-way interaction was not significant (*F*_(1,24)_ = 0.457, *p*_*FDR*_ = 0.809).

In the audiovisual condition, the two-way interaction between attention location and stimulus location was significant (*F*_(1,24)_ = 7.390, *p*_*FDR*_ = 0.032, η_p_^2^ = 0.235). Simple effects analysis revealed that when the stimulus appeared on the left, the amplitude was significantly more negative when attention was directed to the left (*M* = 2.764 μν, *SE* = 0.525) compared to the right (*M* = 3.397 μν, *SE* = 0.569; *p* = 0.024). The main effects of attention location (*F*_(1,24)_ = 1.255, *p*_*FDR*_ = 0.411), electrode location (*F*_(1,24)_ = 6.386, *p*_*FDR*_ = 0.114), and stimulus location (*F*_(1,24)_ = 2.734, *p*_*FDR*_ = 0.222) were not significant. None of the other interactions were significant: attention location × electrode location (*F*_(1,24)_ = 0.127, *p*_*FDR*_ = 0.933), stimulus location × electrode location (*F*_(1,24)_ = 4.138, *p*_*FDR*_ = 0.106), and the three-way interaction (*F*_(1,24)_ = 0.055, *p*_*FDR*_ = 0.934).

In summary, the SN component revealed two key findings. First, a consistent left-space specific attention effect was observed in both auditory and audiovisual conditions. Second, a fundamental difference emerged in sensory processing: a robust contralateral sensory bias was present for auditory stimuli, but this basic organizational principle was abolished when a visual stimulus was concurrently presented.

To examine how the auditory and audiovisual conditions modulate event-related functional connectivity, we compared the attended-unattended wPLI contrasts for each condition (see Figures 2B–2D). Statistical testing revealed that the attention-related connectivity increase was significantly stronger in the auditory condition than in the audiovisual condition, particularly for the FC1-T8 (*p*_*FDR*_ < 0.001) and Cz-T8 (*p*_*FDR*_ < 0.001) electrode pairs (see Figure 2E). These findings indicate that during a unimodal auditory attention task, the brain exhibits enhanced trial-locked, event-related coupling between the frontal (FC1), central (Cz), and right auditory (T8) regions. In contrast, under audiovisual attention, this connectivity enhancement is attenuated, suggesting that multimodal integration may redistribute or dilute the goal-driven attentional modulation observed in the unimodal task. Overall, the time-domain wPLI results show that the attention-related connectivity increase is larger in the auditory condition than in the audiovisual condition, indicating a stronger short-latency modulation of fronto-temporal coupling under the auditory condition.

#### ACOP

**Figure 3:**
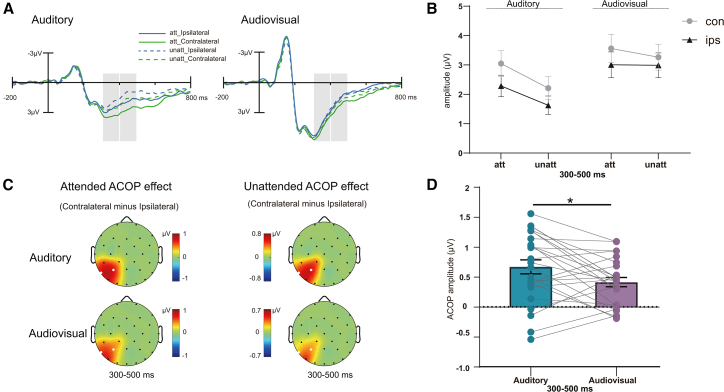
Auditory contralateral occipital positivity (ACOP) as a function of stimulus type and spatial attention (A) Grand-average ERPs time-locked to sound onset for the auditory and audiovisual conditions, shown separately for attended (att) and unattended (unatt) trials and for electrodes contralateral vs. ipsilateral to the sound location. Traces are averaged over the parietal cluster P3/P4/P7/P8. The gray bar marks the 300–500 ms analysis window used to quantify ACOP. (B) Mean amplitudes (μV) extracted from 300 to 500 ms as a function of modality (A vs. AV), attention (att vs. inatt), and laterality (contralateral vs. ipsilateral). Error bars indicate mean ± SEM across participants. (C) Scalp topographies of the contralateral − ipsilateral difference (ACOP effect) for A and AV under att and unatt conditions within 300–500 ms. Warmer colors denote greater contralateral positivity. (D) Participant-wise ACOP amplitudes (contralateral - ipsilateral) at P3/P4/P7/P8 in 300–500 ms for A and AV. Boxes show the mean with SEM (error bar: SEM); dots are individual participants connected across modalities. ∗*p* < 0.05.

A three-way repeated-measures ANOVA with factors of hemisphere (contralateral, ipsilateral), stimulus type (audiovisual, auditory), and spatial attention (attended, unattended) was conducted on mean amplitudes measured over parietal regions (P3/P4, P7/P8) during the 300–400 ms and 400–500 ms time windows (see Table 2; Figures 3A–3C). A significant main effect of hemisphere was observed (300–400 ms: *F*_(1,24)_ = 33.809, *p*_*FDR*_ < 0.001, η_p_^2^ = 0.585, 400–500 ms: *F*_(1,24)_ = 45.238, *p*_*FDR*_ < 0.001, η_p_^2^ = 0.653), with greater positivity over the contralateral compared to the ipsilateral parietal scalp. The main effect of stimulus type was significant during the 300–400 ms interval (*F*_(1,24)_ = 26.562, *p*_*FDR*_ < 0.001 η_p_^2^ = 0.525), reflecting larger amplitudes for audiovisual stimuli, but this effect disappeared in the 400–500 ms interval (*F*_(1,24)_ = 0.368, *p*_*FDR*_ = 0.642). The main effects of spatial attention were significant during 400–500 ms (*F*_(1,24)_ = 7.072, *p*_*FDR*_ = 0.027 η_p_^2^ = 0.228), with responses being larger under attended conditions than under unattended conditions, but not during the 300–400 ms interval (*F*_(1,24)_ = 4.091, *p*_*FDR*_ = 0.341).

**Table 2:** Three-way repeated-measures ANOVA results for ERP components in 300–400 ms and 400–500 ms time windows

	300-400 ms			400-500 ms		
	*F*	*df*	*p*_*FDR*_	*F*	*df*	*p*_*FDR*_
stimulus type	26.562	1,24	<0.001	0.368	1,24	0.642
spatial attention	4.091	1,24	0.341	7.072	1,24	0.027
hemisphere	33.809	1,24	<0.001	45.238	1,24	<0.001
stimulus type × spatial attention	7.247	1,24	0.027	6.696	1,24	0.027
stimulus type × hemisphere	6.219	1,24	0.029	5.102	1,24	0.037
spatial attention × hemisphere	4.833	1,24	0.038	2.550	1,24	0.342
three-way interaction	0.143	1,24	0.708	0.554	1,24	0.642

Notes: The FDR-corrected *p* value was denoted as “*p*_FDR_”.

The interaction between stimulus type and spatial attention was significant for both the 300–400 ms interval (*F*_(1,24)_ = 7.247, *p*_*FDR*_ = 0.027, η_p_^2^ = 0.232) and 400–500 ms interval (*F*_(1,24)_ = 6.696, *p*_*FDR*_ = 0.027, η_p_^2^ = 0.218). Simple effects analysis revealed that under both attended and unattended conditions, the AV condition resulted in a greater response than the A condition during the 300–400 ms interval (all *p* < 0.001). This suggests that regardless of whether the participants focused on a specific location, the neural responses triggered by the audiovisual combined stimuli within the 300–400 ms interval were stronger. Conversely, for the A condition, the attended location elicited a greater response than the unattended location in both the 300–400 ms (*p* = 0.002) and 400–500 ms intervals (*p* = 0.001). The allocation of attention indeed influenced the processing of the auditory stimulus.

The interaction between stimulus type and hemisphere was significant in both windows (300–400 ms: *F*_(1,24)_ = 6.219, *p*_*FDR*_ = 0.029, η_p_^2^ = 0.206; 400–500 ms: *F*_(1,24)_ = 5.102, *p*_*FDR*_ = 0.036, η_p_^2^ = 0.175). Follow-up tests confirmed that the contralateral hemisphere exhibited stronger responses than the ipsilateral hemisphere for both auditory (*p* < 0.001) and audiovisual (*p* < 0.001) stimuli during 300–400 ms and 400–500 ms intervals. Notably, the audiovisual enhancement (AV > A) was more pronounced in the contralateral hemisphere during the 300–400 ms interval (*p* < 0.001) but remained significant ipsilaterally (*p* < 0.001). This may suggest that multisensory integration enhances neural responses not only in specific hemispheres but also exhibits similar enhancement effects across the entire cortical region.

The interaction between spatial attention and hemisphere was significant for the 300–400 ms interval (*F*_(1,24)_ = 4.833, *p*_*FDR*_ = 0.038, η_p_^2^ = 0.168) but not for the 400–500 ms interval (*F*_(1,24)_ = 2.550, *p*_*FDR*_ = 0.342). Simple effects analysis within the contralateral condition showed that the contralateral hemisphere exhibited stronger responses than the ipsilateral hemisphere for both attended (*p* < 0.001) and unattended (*p* = 0.001) conditions. Notably, the attended location elicited a greater response than the unattended location in the contralateral hemisphere during the 300–400 ms interval (*p* = 0.019).

Finally, the three-way interaction between stimulus type, attention location, and hemisphere was not significant (300–400: *F*_(1,24)_ = 0.143, *p*_*FDR*_ = 0.708, η_p_^2^ = 0.006, 400–500 ms: *F*_(1,24)_ = 0.554, *p*_*FDR*_ = 0.642). In the 300–400 ms interval, our research on AV stimuli showed no significant difference in brain activation between contralateral and ipsilateral hemispheres under unattended conditions. However, significant hemispheric differences were observed in both 300–400 ms and 400–500 ms under both attended and unattended conditions for AV and A stimuli, indicating a strong lateralization effect.

Subsequently, to further investigate the influence of spatial attention and stimulus types on lateralization, we conducted a two-way ANOVA with factors of stimulus type (audiovisual, auditory) and spatial attention (attended, unattended) in the parietal region (P3/P4, P7/P8). The results revealed that the main effect of stimulus type was significant (300–500: *F*_(1,24)_ = 6.061, *p*_*FDR*_ = 0.035, η_p_^2^ = 0.202; see Figure 3D), with greater ACOP in auditory conditions. However, the main effect of attentional location was not significant during 300–500 ms (*F*_(1,24)_ = 4.204, *p*_*FDR*_ = 0.341). The interaction was not significant (*F*_(1,24)_ = 0.386, *p*_*FDR*_ = 0.642). The results indicate that ACOP is influenced by task-irrelevant and unnoticed vision. Furthermore, both attended and unattended locations elicited greater positive ERPs in the contralateral hemisphere than in the ipsilateral hemisphere in the endogenous spatial attention condition. The ACOP is a reflection of an automatic process that should not be modulated by endogenous spatial attention.

#### Auditory-to-visual cross-modal attentional spreading

To index cross-modal attentional spreading, we computed a spreading measure defined as [(AV-A)-V] for the attended and unattended conditions. Cross-modal spreading was operationally defined as a significant difference between these two conditions. Paired-sample *t* tests were conducted in consecutive 100-ms windows from 200 to 700 ms at the centro-parietal ROI (Pz/P3/P4, CP1/CP2) (see Table 3 for detailed statistics). A significant attentional spreading effect emerged in the 500–600 ms window, with greater negative amplitude in the attended than in the unattended condition (*t*_(24)_ = −2.233, *p*_*FDR*_ = 0.046, *d* = −0.446). No reliable attention-related differences were observed in the remaining windows (200–300 ms: *t*_(24)_ = 0.140, *p*_*FDR*_ = 0.889; 300–400 ms: *t*_(24)_ = −1.464, *p*_*FDR*_ = 0.234; 400–500 ms: *t*_(24)_ = −1.928, *p*_*FDR*_ = 0.176; 600–700 ms: *t*_(24)_ = −1.820, *p*_*FDR*_ = 0.176).

**Table 3:** Paired-sample *t* test results on centro-parietal and occipital ERP amplitudes (200–700 ms in 100 ms Intervals)

TW	Centro-Parietal		Occipital	
	*t*	*p*_*FDR*_	*T*	*p*_*FDR*_
200–300	0.140	0.889	−0.612	0.655
300–400	−1.464	0.234	−2.528	0.046
400–500	−1.928	0.176	−2.092	0.047
500–600	−2.233	0.047	−2.358	0.046
600–700	−1.820	0.176	−1.779	0.176

Notes: TW (time window, ms).

Applying the same definition of spreading, we compared spreading indices between attended and unattended conditions at the occipital ROI (Oz/O1/O2, PO7/PO8). A significant attentional spreading effect was observed across 300–600 ms, with greater negative amplitude in the attended condition than in the unattended condition: 300–400 ms: *t*_(24)_ = −2.528, *p*_*FDR*_ = 0.046, *d* = −0.506; 400–500 ms: *t*_(24)_ = −2.092, *p*_*FDR*_ = 0.047, *d* = −0.418; 500–600 ms: *t*_(24)_ = −2.358, *p*_*FDR*_ = 0.046, *d* = −0.472. No significant differences were found in the 200–300 ms or 600–700 ms time windows (200–300 ms: *t*_(24)_ = −0.612, *p*_*FDR*_ = 0.655; 600–700 ms: *t*_(24)_ = −1.779, *p*_*FDR*_ = 0.176).

## Discussion

In this ERP study, we manipulated stimulus type (auditory, audiovisual) and spatial attention (attended, unattended) to examine how reciprocal audiovisual interactions operate temporally and asymmetrically. Neural measures revealed a reciprocal but asymmetric pattern of crossmodal interactions. Initial processing phases were dominated by automatic, stimulus-driven visual-to-auditory suppression, reflected in reduced ACOP, and attenuated auditory selection negativity (SN), consistent with competition for limited neural resources. Subsequent phases showed auditory-to-visual facilitation, reflected in a sustained negative difference wave (extracted visual minus visual-only) that was larger for attended than unattended auditory streams, indicating goal-driven cross-modal attentional spreading. These distinct but complementary effects suggest a division of labor in crossmodal attention, whereby initial stimulus-driven suppression and subsequent goal-driven facilitation jointly shape multisensory processing. The following sections elaborate on the mechanisms underlying the suppressive and facilitative interactions observed.

### Initial stimulus-driven suppression of auditory processing by task-irrelevant visual input

Behavioral results showed that when participants selectively attended to the auditory modality, response time and accuracy did not significantly differ between auditory and audiovisual targets, indicating minimal visual interference at the behavioral level. This finding is consistent with previous crossmodal studies demonstrating that visual distractors can elicit neural responses without significantly impairing auditory task performance.^40^^,^^41^^,^^42^ We acknowledge that the high correct response rates (>97%) across conditions indicate that the auditory task was not particularly challenging for participants. This ceiling effect in behavioral performance likely reduced our sensitivity to detect subtle behavioral manifestations of crossmodal interference. This pattern is fully consistent with perceptual load theory.^43^^,^^44^ When a primary task consumes minimal attentional resources, surplus capacity automatically spills over to process task-irrelevant stimuli, yet without necessarily impairing behavioral performance on the primary task. Thus, the lack of a significant behavioral difference between A and AV trials can be parsimoniously explained by this low perceptual load, which allowed sufficient resources for both accurate task performance and the processing of the visual stimulus.

In contrast, our ERP results delineated a distinct neurophysiological signature of auditory spatial attention, characterized by a stable left-space-specific attention effect and a robust contralateral organization of early sensory processing. These patterns are consistent with prior reports that goal-driven attentional mechanisms sharpen auditory stream segregation and enhance target detection.^27^^,^^45^^,^^46^^,^^47^ More importantly, the present data provide strong new evidence for the engagement of a right hemisphere-dominant network during auditory spatial attention. It is well established that the right hemisphere plays a superior role in spatial monitoring and sustained vigilance across both hemifields.^48^^,^^49^^,^^50^ This inherent functional asymmetry offers a parsimonious account for our findings: the most pronounced electrophysiological signatures emerged selectively for left-sided stimuli—the primary receptive field of the attention-dominant right hemisphere. The observed contralateral sensory bias further indicates that this hemisphere notably orchestrates early auditory processing, particularly for stimuli appearing in the left space. Together, these results reinforce the view that the neural architecture of spatial attention is intrinsically biased, with the right hemisphere serving as the core of a lateralized network for auditory spatial processing.^51^^,^^52^

Critically, when concurrent visual stimuli were introduced, the previously robust contralateral sensory bias was eliminated, and frontal-auditory connectivity among frontal (FC1), central (Cz), and right auditory (T8) regions was markedly reduced. We propose that this reflects competitive interplay for shared neural resources,^53^^,^^54^^,^^55^ wherein task-irrelevant visual inputs recruit overlapping frontoparietal networks,^56^^,^^57^ thereby diminishing the resources available for maintaining the specialized lateralized architecture of unimodal auditory processing. In the audiovisual condition, the observed reduction in contralateral bias likely results from this competitive shift in attentional weighting,^54^^,^^57^ where automatic processing of visual stimuli redirects computational resources toward a more distributed, multimodal representation. This resource redistribution underscores the capacity of even task-irrelevant vision to competitively bias cortical processing streams, accounting for the diminished lateralization of auditory responses.^58^^,^^59^^,^^60^^,^^61^

The observed pattern, consisting of left-space specific neurophysiological effects and right-lateralized functional connectivity, provides compelling evidence for the involvement of a right-hemisphere-dominant network in auditory spatial attention. This network integrates the right hemisphere’s dual roles in spatial attentional control^49^^,^^62^ and the processing of complex auditory spatial information.^63^ During unimodal auditory attention, this specialized network is optimally engaged, resulting in clear neural signatures for left-side stimuli, manifested in enhanced connectivity within core fronto-auditory circuits (FC1-Cz-T8).^64^^,^^65^

Converging evidence came from ACOP, an index of auditory-driven visual cortical activation, which was consistently reduced in audiovisual compared to auditory-only conditions, independent of spatial attention. This suggests that irrelevant visual input can automatically suppress auditory-driven visual cortical responses, likely via early-stage multisensory competition.^17^^,^^66^^,^^67^ Prior studies have shown that visual information can modulate early occipital activity elicited by sound, either by enhancing spatial alignment^18^ or interfering with attention when incongruent.^14^ Such suppression can occur even without spatial or semantic conflict, reflecting automatic modality competition under limited neural resources.^35^^,^^68^

In addition, a principled account of ACOP suppression comes from divisive normalization, a canonical computation by which multisensory signals are integrated through gain-control operations.^20^^,^^69^^,^^70^ In this framework, visual input reduces the effective gain of auditory-driven activity in the visual cortex, yielding sub-additive or suppressive responses under multisensory competition.^20^^,^^71^^,^^72^ This mechanism predicts ACOP attenuation regardless of whether suppression reflects active inhibition or passive nonlinear saturation because both arise from divisive scaling. The ACOP reduction observed here therefore aligns with normalization-based models of multisensory processing, which propose that combined inputs are integrated through divisive rather than linear operations.

Beyond this mechanistic account of attentional modulation, our findings also raise a broader question about ACOP’s role in spatial perception under more ecologically valid, conflicting conditions. Importantly, we observed that even a spatially neutral, task-irrelevant visual stimulus could suppress the auditory-evoked ACOP response. This demonstrates that crossmodal competition in the visual cortex occurs even in the absence of spatial discrepancy.^73^^,^^74^^,^^75^ This foundational competition may serve as a neural precursor to more pronounced perceptual phenomena, such as the ventriloquism effect, where a spatially discrepant visual stimulus robustly biases auditory localization.^76^^,^^77^^,^^78^^,^^79^ It is therefore a compelling and relevant question for future research to investigate whether the suppressive interaction we identified is amplified or otherwise modulated when visual and auditory inputs are spatially misaligned and whether ACOP dynamics can directly predict the resulting perceptual bias. Future studies that systematically vary spatial alignment while recording ACOP and behavioral localization will be crucial to unravel how early crossmodal competition, as indexed by ACOP, evolves into perceptual binding or recalibration.

Together, these mechanisms provide a comprehensive account of how even behaviorally irrelevant visual stimuli can alter the neural representations underlying auditory spatial processing and its downstream consequences. The combination of resource competition, modality bias, and canonical multisensory normalization explains why the contralateral sensory bias (SN) and fronto-auditory connectivity observed in auditory-only conditions are reduced under audiovisual presentation and why ACOP is automatically attenuated regardless of attentional state. These results highlight that stimulus-driven crossmodal interactions are robust and largely automatic, revealing the limits of behavioral measures in capturing subtle neural interference.

### Subsequent goal-driven auditory-to-visual attentional spreading

In contrast to the suppressive visual influence observed in stimulus-driven processing, we found robust evidence for a goal-driven enhancement of visual cortical responses by auditory spatial attention. This effect showed clear attention-dependent facilitation, emerging at occipital sites from 300 to 600 ms and at centro-parietal sites from 500 to 600 ms. The presence of this effect only when the auditory stream was attended supports a genuine crossmodal attentional spreading mechanism from audition to vision, consistent with prior work demonstrating visual-to-auditory spreading to the reverse pathway.^21^^,^^24^^,^^25^^,^^29^^,^^30^^,^^31^^,^^32^^,^^34^^,^^80^ This spatiotemporal profile demonstrates that auditory-to-visual spreading depends critically on the attentional state of the auditory input.

The most parsimonious explanation, consistent with prior accounts emphasizing supramodal control of multisensory attention,^81^^,^^82^ is that auditory attention recruits frontoparietal networks that may modulate visual cortices via feedback projections. While our connectivity analysis showed strengthened coupling between the frontal (FC1) and auditory (T8) regions during auditory selection, no significant connectivity effects were observed in the visual cortices. This suggests that changes in visual cortical activity may be driven by top-down feedback from attention networks rather than through direct connectivity with the visual cortex. Once engaged for auditory stream segregation, these same control networks may extend their influence to the visual system, enhancing the processing of visual input, even when it is spatially neutral and irrelevant to the task. This interpretation aligns with frameworks that highlight the role of dorsal attention networks in multisensory processing coordination.^83^^,^^84^^,^^85^^,^^86^

We further considered the potential contribution of temporal binding between auditory and visual inputs. The precise synchrony of our stimuli likely promoted their integration into a coherent audiovisual representation,^80^^,^^87^ and within such a bound representation, attention to the auditory component could plausibly “spill over” to its visual counterpart.^88^^,^^89^ However, if such spillover were an automatic consequence of temporal binding, a comparable facilitative effect should have been evident even when the auditory stream was unattended. Our data contradict this prediction. Cross-modal facilitation, operationalized as the difference [(AV-A)-V] in the complex between attended and unattended conditions, was strictly dependent on auditory attention and was expressed as sustained modulation over the occipital cortex from 300 to 600 ms. This late-onset, attention-dependent modulation is inconsistent with the early, automatic processes typically associated with temporal binding.^90^ Instead, it aligns with the view that temporal binding creates a perceptual substrate that enables cross-modal interaction, but the actual manifestation of facilitation is gated by goal-directed, supramodal attention, likely implemented via feedback projections from frontoparietal control networks to the visual cortex.^56^^,^^81^

Importantly, the spatiotemporal profile of the crossmodal attention spreading effect highlights its controlled, goal-driven nature. Whereas ACOP reflected stimulus-driven multisensory co-activation, the sustained negative difference ERP was selectively enhanced under spatially attended conditions, consistent with the goal-driven engagement of dorsal attention networks projecting to visual cortices.^82^^,^^84^^,^^87^ This distinction aligns with neurocognitive models proposing that early multisensory integration is largely automatic, while later ERP components capture modality-specific attentional prioritization.^38^^,^^81^ Within this framework, our findings reveal a clear temporal asymmetry: visual-to-auditory influences occur initially and suppressively, whereas auditory-to-visual influences emerge subsequently and facilitatively. This asymmetry can be understood from two complementary perspectives. According to the modality appropriateness framework, vision exerts a stronger stimulus-driven influence over spatial processing.^91^^,^^92^^,^^93^ At the same time, the hierarchical organization of sensory pathways suggests that vision has more direct access to spatial attention networks, whereas auditory modulation is mediated via higher-order integration hubs.^94^^,^^95^ Together, these accounts converge to explain why crossmodal interactions are both reciprocal and asymmetric.

These findings demonstrate a dual pattern of crossmodal interactions: initial visual inputs exert automatic, suppressive influences on auditory spatial processing, whereas subsequent auditory attention facilitates visual processing in an attention-dependent manner. Building on these findings, we propose a descriptive, temporally differentiated framework that conceptualizes these interactions as an initial competitive phase followed by a delayed facilitative phase. While this competitive-facilitative framework is primarily descriptive, it offers a valuable conceptual lens through which to reinterpret and constrain existing models of audiovisual interaction. First, it refines models of early integration by providing empirical evidence that the initial crossmodal interaction is not uniformly integrative^96^^,^^97^ but is instead competitive. This observation challenges the assumption that crossmodal interactions are always facilitative from the outset. The competitive nature of early processing may be governed by canonical neural computations, such as divisive normalization, which regulate the gain of concurrent sensory inputs. Thus, our framework serves as a phenomenological constraint, suggesting that any complete account of early multisensory processing must incorporate a temporally specific competitive component.

Second, the framework enhances the supramodal theory of attention^82^^,^^98^ by documenting that top-down attentional control can initiate a distinct, delayed facilitative phase, operating on a different temporal scale than the initial automatic competition. This dual-phase dynamic (competitive suppression followed by attentional facilitation) adds temporal specificity to the theory, indicating that supramodal attention’s influence is not monolithic but unfolds in a sequenced manner over time. Ultimately, the present descriptive framework highlights a critical empirical pattern: the temporal asymmetry between competitive and facilitative crossmodal interactions. This pattern establishes a clear constraint that future computational and neurobiological models must account for. Specifically, it calls for future work to link these distinct temporal phases to their underlying neural computations (e.g., investigating whether early competition arises from mechanisms such as divisive normalization and how late facilitation is implemented within fronto-parietal attention networks) and to test their causal necessity through targeted interventions.

In conclusion, we manipulated stimulus type (auditory vs. audiovisual) and spatial attention (attended vs. unattended) to examine how reciprocal audiovisual interactions operate temporally and asymmetrically. Our multi-method analyses revealed a clear dissociation between stimulus-driven suppression of auditory processing (reduced SN, ACOP, and fronto-temporal connectivity) and goal-driven facilitation of visual processing (sustained negative difference wave and enhanced parietal activation) by auditory attention. These results demonstrate that audiovisual interactions are reciprocal yet asymmetric, with initial suppression preceding subsequent facilitation. By integrating stimulus-driven visual dominance with goal-driven auditory facilitation, our findings support a descriptive competitive-facilitative framework of crossmodal attention. This framework advances models of multisensory integration by delineating a critical temporal asymmetry that must be accounted for. More broadly, our findings underscore the flexibility of attentional control and provide a foundation for future research into how such asymmetric mechanisms generalize across tasks, spatial contexts, and populations.

### Limitations of the study

Several limitations warrant caution. One important feature of our experimental design was the introduction of a systematic spatial mismatch between the lateralized auditory targets and the central visual stimulus. This configuration was intentionally employed to minimize bottom-up, spatially specific integration and to thereby more cleanly isolate the distinct contributions of automatic, stimulus-driven competition from goal-directed, top-down attentional spreading. Consequently, our findings most directly elucidate the neural dynamics of crossmodal interactions under conditions of spatial disparity, which are ecologically prevalent (e.g., listening to a speaker on one side while viewing a central screen). An important direction for future research will be to determine how the distinct temporal mechanisms identified here—early suppressive competition and late facilitative spreading—are modulated when audiovisual stimuli are spatially aligned.

Another consideration is the salience of our task-irrelevant visual stimuli. While the high-contrast grating was effective in driving visual cortical responses and engaging in crossmodal competition, its salience was likely within a moderate range. This is supported by the absence of significant behavioral interference on auditory task performance, suggesting that the stimuli were not so salient as to completely override the goal-directed auditory attention. Future studies could systematically manipulate visual salience (e.g., luminance, contrast, or motion) to examine how the strength of stimulus-driven suppression scales with the bottom-up salience of the irrelevant input. Finally, while our ERP findings provide strong evidence for the timing and scalp topography of auditory-to-visual attentional spreading, the limited spatial resolution of the technique prevents precise localization of its neural sources. The observed late and sustained modulation likely arises from a distributed network, potentially involving feedback projections from frontoparietal attention areas to visual cortices. However, the exact anatomical generators cannot be conclusively identified from scalp potentials alone. To address this limitation, future research should incorporate neuroimaging methods with higher spatial resolution, such as functional MRI or simultaneous EEG-fMRI, to more precisely map the neural circuits, including contributions from the prefrontal, parietal, and visual cortices, responsible for this supramodal attentional effect. Integrating both temporal and spatial evidence will provide a more comprehensive understanding of how attention mediates cross-modal interactions.

## Resource availability

### Lead contact

Requests for further information and resources should be directed to and will be fulfilled by the lead contact, Jiaying Sun (sunjy_1994@163.com).

### Materials availability

This study did not generate new unique reagents.

### Data and code availability

All data reported in this paper will be shared by the lead contact upon request.
Any additional information required to reanalyze the data reported in this paper is available from the lead contact upon request.

## Acknowledgments

This work was supported by the 10.13039/501100018617Liaoning Revitalization Talents Program (grant no. XLYC2403186, X.T.), the Key Teacher Project of Education Science Research by Liaoning Province (grant 2417, X.T.), and the Zhejiang Provincial Philosophy and Social Sciences Planning Project (grant no. 24NDON135YBM, S.L.).

## Author contributions

Conceptualization, software, data curation, formal analysis, writing-original draft, and writing-review & editing, X.T.; Conceptualization, data curation, formal analysis, writing-original draft, and writing-review & editing, T.Z.; Conceptualization, formal analysis, investigation, supervision, project administration, and writing-review & editing, J.S.; Conceptualization, formal analysis, investigation, supervision, project administration, and writing-review & editing, S.L.

## Declaration of interests

The authors declare no competing interests.

## STAR★Methods

### Key resources table

**Table:** 

REAGENT or RESOURCE	SOURCE	IDENTIFIER
**Software and algorithms**		

MATLAB R2016b	MathWorks	https://www.mathworks.com/products/matlab.html; RRID: SCR_001622
G∗Power 3.1.9.7	Heinrich Heine University Düsseldorf	http://www.gpower.hhu.de; RRID: SCR_013726
E-Prime 3.0	Psychology Software Tools	https://www.pstnet.com/eprime.cfm; RRID: SCR_009567
Brain Vision recorder 2.0	Brain Products GmbH	https://www.brainproducts.com/productdetails.php?id=20; RRID: SCR_016331
EEGLAB 14.0	Swartz Center for Computational Neuroscience	https://sccn.ucsd.edu/eeglab/index.php; RRID: SCR_007292
ERPLAB 6.3.1	UC Davis Center for Mind and Brain	https://erpinfo.org/erplab; RRID: SCR_009574
SPSS 26.0	IBM Corporation	https://www.ibm.com/products/spss-statistics; RRID: SCR_002865

### Experimental model and study participant details

We conducted an *a priori* power analysis using G∗Power 3.1.9.7. The calculation was based on the effect size (η_p_^2^ = 0.16, converted to f ≈ 0.40) reported in a key reference study employing a similar crossmodal attention paradigm.^21^ With an alpha level of 0.05 and a desired power of 0.90 for a within-subjects ANOVA, the analysis indicated a minimum required sample size of 19 participants. Thus, twenty-five participants (10 males and 15 females) without any history of neurological or psychiatric illness participated in the experiment. All participants with normal or corrected-to-normal vision were right-handed. All participants provided informed consent before the study according to the Declaration of Helsinki. This study was approved by the Ethics Committee of Liaoning Normal University. Participants were all paid for their participation.

### Method details

#### Apparatus, stimuli, and design

The experiment was conducted in a dimly lit room. The visual stimuli were presented on a 23.8-in. A PHILIPS monitor (242EIGSJ, resolution 1,024 × 768, refresh rate 60 Hz) was used. The stimuli were scripted using "E-prime" software (version 3.0, Psychology Software Tools, Sharpsburg, PA, USA). The visual (V) stimuli were presented on a black background display (0.4 cd/m2). Participants sat in front of the monitor at a viewing distance of approximately 60 cm and were required to fix their eyes on a white (155.2 cd/m2) fixation cross (0.5 ° × 0.5 ° of visual angle). The auditory (A) target stimulus was white noise, while the auditory non-target stimulus produced by SoundEngine was a 1000 Hz pure tone (65 dB, 100 ms, rising and falling time of 10 ms). The task-irrelevant visual stimulus was a white horizontal square wave grating (4 ° × 4 °; the spatial frequency was 1 cycle/degree). The audiovisual (AV) stimulus consisted of the simultaneous presentation of both visual and auditory stimuli.

A 2 (spatial attention: attended, unattended) × 2 (stimulus type: audiovisual, auditory) within-subject design was adopted in the present experiment. The flow of a single trial in the experimental phase is shown in Figure 1. In each trial, participants were instructed to attend to the left side or the right side covertly. The auditory target stimulus was presented from left/right speakers at 33 ms, and the intertrial interval was 950–1,050 ms. Visual stimuli were presented at the bottom of the screen (vertical distance: 6°). The target stimulus accounted for 10% of all stimuli. The experiment contained 12 blocks for a total of 1200 trials (half attended to the left side, half attended to the right side). Under each combination condition, there were equal numbers of audiovisual and auditory stimuli. The proportions of various types of stimuli were as follows: visual stimuli 16%, auditory stimuli 42%, and audiovisual stimuli 42%. At the end of each block, a summary of their performance (the number of correct responses) was displayed on the screen, and participants were allowed a short break between blocks. The experiment required participants to ignore the visual stimuli presented either simultaneously with the auditory stimuli in the center or separately, paying attention only to the auditory stimuli on one side of the screen (attended location) and ignoring any stimulus that appeared on the other side of the screen (unattended location). Participants were instructed to press the 'B' key as quickly and accurately as possible upon detecting an auditory target stimulus at the attended location. This auditory target could be presented either in isolation or accompanied by a simultaneous, task-irrelevant visual stimulus.

#### Electrophysiological recording and preprocessing

EEG data were recorded by the German Brain Product workstation, which was used to record EEG signals using 32 electrodes mounted on an electrode cap as specified by the International 10–20 system, and an EEG system (Brain Products, Brain Vision Recorder 2.0) was used to record ERP signals. The unilateral (left) earlobe was used as the reference electrode, and a ground electrode was placed on the medial frontal aspect. Vertical eye movements and blinks were detected by a pair of bipolar electrodes (Fp1, Fp2) placed above the eyes, while the right earlobe was used as the reference electrode.

The raw signals were digitized with a sampling frequency of 500 Hz, and all impedances were kept below 5 kΩ. EEGLAB (version 14.0b) and ERPLAB (version 6.3.1) software were used to filter the stored continuous data after 0.1 Hz high-pass and 30 Hz low-pass (slope = 24 dB/octave) filtering. Based on the previous literature, the crossmodal spread of the attention effect occurs in the period of 200–700 ms. Therefore, the continuous EEG signals were then segmented into 800 ms epochs that were time-locked to the stimulus onset with a 200 ms pre-stimulus baseline and were baseline-corrected. It should be noted that automatic artifact rejection was performed based on a threshold of ±80 μv for both the EEG and EOG electrodes to eliminate epochs contaminated by horizontal eye movements, eye blinks, and muscle activities. Next, the segmented data without artifacts are classified and superimposed according to the experimental conditions. The grand average ERP was computed across all participants for each experimental condition. The superposition ERP of each subject under different conditions was obtained.

The remaining EEG epochs, sorted according to spatial attention (attended, unattended) and stimulus type (audiovisual, auditory), were averaged to obtain corresponding ERP waveforms. EEG preprocessing and subsequent ERP analysis were performed using the EEGLAB toolboxes^99^ in combination with custom-built MATLAB scripts (The MathWorks, Inc.).

Thus, there were on average 174.80 ± 3.59 (M ± SE) valid epochs in the V non-target condition, 174.64 ± 3.51 in the A attended non-target condition, 172.88 ± 3.76 in the An unattended non-target condition, 173.04 ± 3.74 in the AV attended non-target condition, and 174.44 ± 3.92 in the AV unattended non-target condition.

### Quantification and statistical analysis

EEG preprocessing and ERP quantification were performed using EEGLAB (v14.0b) and ERPLAB (v6.3.1) and custom MATLAB scripts (MATLAB R2016b). Statistical analyses were performed using IBM SPSS Statistics (v26.0). Unless otherwise stated, n denotes the number of independent participants included in each analysis (*n* = 25). The exact n, statistical tests, test statistics, degrees of freedom, *p* values (including *p*FDR where applicable), and effect sizes are reported in the Results and figure legends. Data are summarized as mean ± SEM unless otherwise specified. All tests were two-tailed with α = 0.05.

#### Behavioral data analysis

The accuracy (ACC) and reaction time (RT) of the participants were calculated between 100 ms and 1,000 ms post-stimulus. Subsequently, a paired sample *t* test was performed on the accuracy and response time of the audiovisual and auditory targets in the attended location.

#### ERP data analysis

To collapse ERP data for visualization and statistical analysis, the regions and time intervals of interest were separately selected for the ERP components of interest, namely, the spatial attention effect, the crossmodal attentional spreading, and the auditory-evoked contralateral occipital positivity (ACOP), as described below. Data from trials with incorrect responses were excluded from all the ERP analyses. For ERP preprocessing, epochs were time-locked to the sound onset.

#### Spatial attention effect analysis

To examine whether participants maintained their space-based auditory selective attention as instructed, the spatial attention effect was calculated.^27^ To address potential concerns regarding the representativeness of the analyzed non-target trials and the confounding effects of response suppression, we conducted supplementary control analyses. These confirmed the validity of our approach by demonstrating that: (1) response-related activity (P300) was dissociable from the effects of interest (See Figure S1; Table S1), and (2) the spatial attention effect on the sustained negativity (SN) component did not differ between target and non-target trials (See Table S2). The mean amplitude of the SN component was quantified within a 220–320 ms time window post-stimulus at a fronto-central region of interest (ROI). This time window and ROI were selected because the auditory spatial attention SN is typically observed as a sustained negativity maximal over fronto-central scalp sites during this latency range.^27^^,^^100^^,^^101^ The ROI comprised the following electrodes: FC1, FC2, FC5, FC6, F3, F4, C3, and C4. Separate three-way repeated-measures ANOVAs were performed for the auditory and audiovisual conditions, with factors of attention location (left vs. right), stimulus location (left vs. right), and electrode location (left vs. right). For this analysis, the ROI electrodes were averaged into left (FC1, FC5, F3, C3) and right (FC2, FC6, F4, C4) hemisphere values. This design allowed us to dissociate sensory processes, driven by the physical location of the stimulus, from top-down control processes, driven by the directed focus of attention, and to examine the contralateralization pattern of each. When the sphericity assumption was violated, Greenhouse-Geisser correction was applied. Multiple comparisons were controlled using the false discovery rate (FDR) procedure^102^; FDR-adjusted *p* values are reported as *p*FDR.

#### Functional connectivity analysis

To examine event-related functional interactions between brain regions during auditory and audiovisual attention, we computed the weighted phase lag index (wPLI) in the time domain. Specifically, ERP components within the non-target time windows were extracted, and the instantaneous phase differences between electrode pairs were calculated across trials using the Hilbert transform. This time-domain wPLI approach captures trial-locked, event-related phase synchronization, reflecting dynamic functional connectivity during attentional processing rather than ongoing oscillatory activity. While time-domain wPLI effectively captures short-latency, event-related connectivity dynamics, it does not resolve frequency-specific oscillatory interactions. Thus, the results should be interpreted as reflecting temporal, stimulus-locked functional coupling rather than frequency-band specific synchronization.

We quantified connectivity differences between conditions as follows: (1) "attended auditory" vs. "unattended auditory" trials in the auditory-only task and (2) "attended audiovisual" vs. "unattended audiovisual" trials in the audiovisual task. Pairwise comparisons of wPLI differences between these tasks were performed using two-tailed paired-sample *t* tests. To control for multiple comparisons, all *p* values were corrected using the false discovery rate (FDR) procedure (*p*FDR < 0.05).

#### ACOP analysis

First, ERP waveforms were collapsed across the auditory stimulus location (left/right) and electrode location (left/right) to obtain ERPs recorded on the contralateral and ipsilateral hemispheres. The three-way repeated-measures ANOVA with factors of 2 (hemisphere: contralateral, ipsilateral) × 2 (stimulus type: audiovisual, auditory) × 2 (spatial attention: attended, unattended) were analyzed as the mean amplitude difference over two pairs of parietal electrode sites (P3/P4, P7/P8) within the time windows of 300–400 and 400–500 ms post-stimulus onset. These time windows and electrode sites were selected based on prior literature characterizing the ACOP component, which typically manifests as a positive deflection over parietal regions during these latencies.^18^^,^^103^

Subsequently, to investigate the differences in occipital activation induced by auditory stimuli across varying stimulus types and spatial attention conditions, we performed a two-way ANOVA with factors of 2 (spatial attention: attended, unattended) × 2 (stimulus type: audiovisual, auditory), using the mean differences between contralateral and ipsilateral conditions.

#### Crossmodal attentional spreading analysis

**Figure 4:**
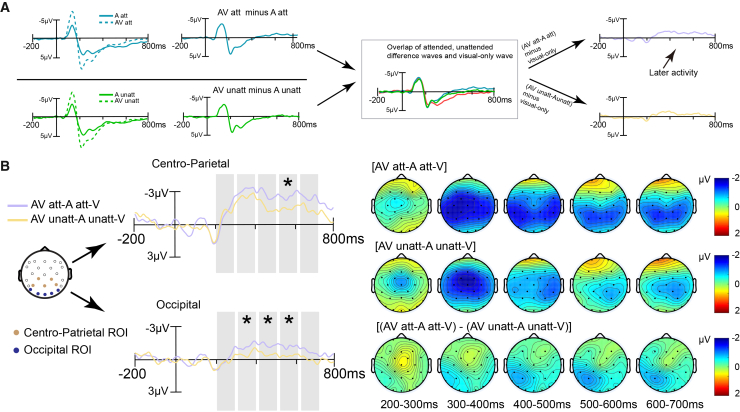
Neural evidence for the auditory-to-visual crossmodal attentional spreading (A) The visual component was isolated by subtracting the auditory ERP from the audiovisual ERP at both attended and unattended locations. Difference waveforms were then obtained by subtracting the visual-only ERP from these extracted visual components. (B) Grand-average difference in ERP waves at centro-parietal and occipital ROIs. Shaded areas indicate the five consecutive 100 ms time windows (200–700 ms) used for amplitude analysis. Scalp topographies show the difference waves between extracted-visual and visual-only conditions for attended [AV _attended_-A _attended_-V] and unattended [AV _unattended_-A _unattended_-V] conditions, as well as the topographies of their direct difference, across each time window. (∗*p* < 0.05).

To investigate the auditory-to-visual crossmodal attentional spreading effect, we first isolated the visual component in both attended and unattended conditions by subtracting the auditory-only ERP from the audiovisual ERP (i.e., AV _attended_ - A _attended_ and AV _unattended_ - A _unattended_). We then derived a crossmodal spreading measure by further subtracting the visual-only ERP from these extracted visual components for each attentional condition, resulting in the difference waves: AV _attended_ - A _attended_ - V and AV _unattended_ - A _unattended_ - V (Figure 4A).

To directly quantify and test for the presence of crossmodal attentional spreading, paired-sample *t* tests were conducted to compare the mean amplitude of the crossmodal spreading measure between the attended and unattended conditions across consecutive 100-ms time windows between 200 and 700 ms post-stimulus.^21^^,^^26^^,^^29^^,^^31^^,^^32^ The significant negative difference in amplitude between the attended and unattended conditions in an ROI consisting of the centro-parietal (Pz, P3, P4, CP1, CP2) and occipital region (PO7, PO8, Oz, O1, O2) during the 200–700 ms was used as an index to measure the crossmodal attentional spreading.
