# Supplementary material for: Echoes of the mind’s eye: Reciprocal crossmodal interaction between auditory and visual processing
Source: iScience. 2026 Feb 12;29(3):114990. doi: 10.1016/j.isci.2026.114990 (PMC12955579; doi:10.1016/j.isci.2026.114990)
Supplement: Document S1. Figure S1, Tables S1 and S2 [file mmc1.pdf]

iScience, Volume *29*

Supplemental information

Echoes of the mind's eye: Reciprocal crossmodal interaction between auditory and visual processing

Xiaoyu Tang, Ting Zhang, Jiaying Sun, and Sa Lu

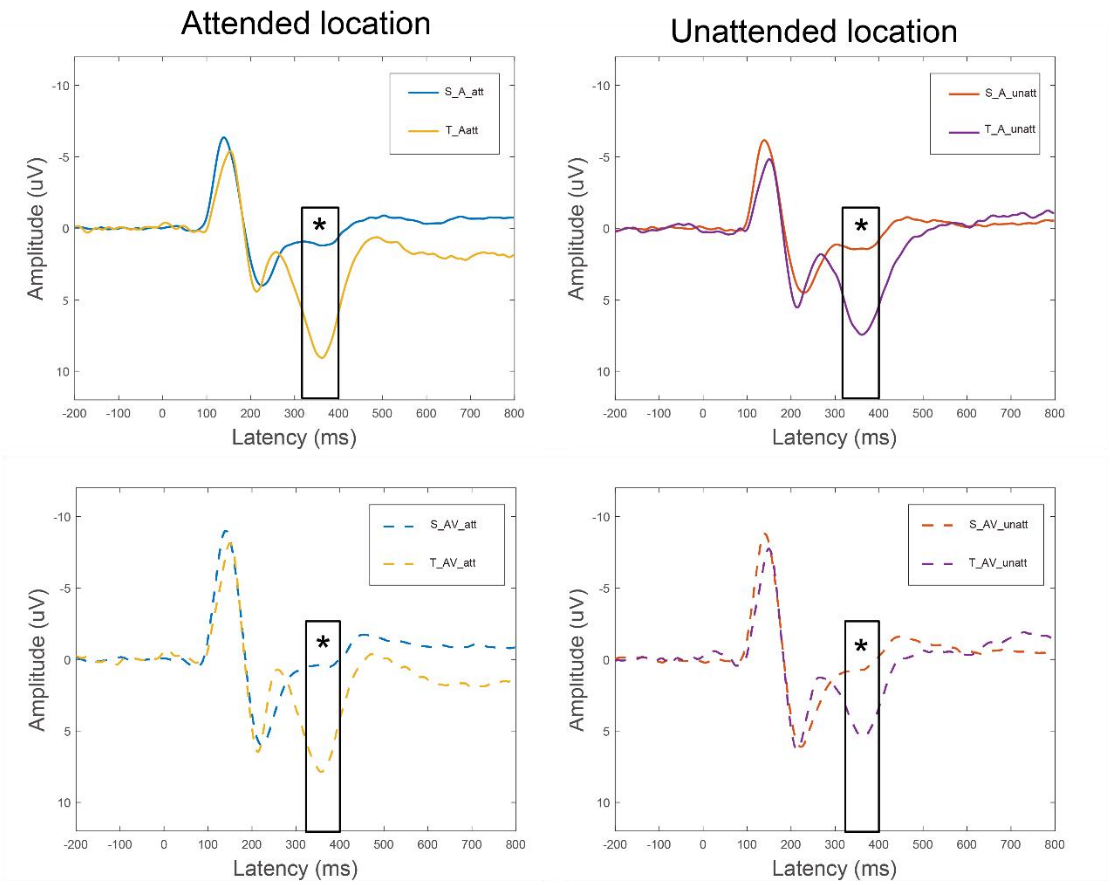

Figure S1. The figure displays event-related potentials recorded at frontal electrodes (FC1, FC2, FC5, FC6, F3, F4, Fz) in the 330–400 ms time window. The four panels contrast the P300 responses to target and standard stimuli under each condition: attended auditory (top-left), unattended auditory (top-right), attended audiovisual (bottom-left), and unattended audiovisual (bottom-right).

Table S1.Three-Way Repeated Measures ANOVA Results for P300 Amplitudes (330–400 ms)

	F	df	p_{FDR}
stimulus type	58.326	1,24	<0.001
target condition	42.635	1,24	<0.001
spatial attention	9.763	1,24	0.007
stimulus type×target condition	10.029	1,24	0.007
spatial attention×stimulus type	1.388	1,24	0.292
spatial attention×target condition	17.746	1,24	0.002
three-way interaction	0.636	1,24	0.433

Note: The FDR-corrected p value was denoted as "p_{FDR}".

Table S2. Results of the Four-Way Repeated-Measures ANOVA on SN Amplitudes in the Auditory and Audiovisual Conditions

	Auditory		Audiovisual	
	F	p_{FDR}	F	p_{FDR}
attention location	0.776	0.725	0.352	0.962
stimulus location	0.027	0.970	3.496	0.277
electrode location	3.606	0.525	5.432	0.145
target condition	1.867	0.608	0.119	0.962
target condition×electrode location	1.570	0.608	0.418	0.524
target condition×attention location	0.007	0.970	0.036	0.851
electrode location×attention location	0.237	0.788	0.018	0.893
attention location×stimulus location	0.435	0.788	0.012	0.914
target condition×stimulus location	0.001	0.970	0.054	0.819
electrode location×stimulus location	17.752	0.001	15.133	0.007
target condition×electrode location×stimulus location	1.378	0.608	7.255	0.045
target condition×electrode location×attention location	0.249	0.788	0.242	0.893
target condition×attention location×stimulus location	1.201	0.608	2.625	0.354
electrode location×attention location×stimulus location	0.254	0.789	0.002	0.962
Four-way interaction	1.577	0.609	0.096	0.759

Notes: The FDR-corrected p value was denoted as “p_{FDR}”.
